# One-Stage Multi-Sensor Data Fusion Convolutional Neural Network for 3D Object Detection

**DOI:** 10.3390/s19061434

**Published:** 2019-03-23

**Authors:** Minle Li, Yihua Hu, Nanxiang Zhao, Qishu Qian

**Affiliations:** 1 State Key Laboratory of Pulsed Power Laser Technology, College of Electronic Engineering (National University of Defence Technology), Hefei 230037, China; southfly@163.com (N.Z.); creamy777@163.com (Q.Q.); 2 Anhui Province Key Laboratory of Electronic Restriction Technology, Hefei 230037, China

**Keywords:** multi-sensor, object detection, convolutional neural network, point cloud

## Abstract

Three-dimensional (3D) object detection has important applications in robotics, automatic loading, automatic driving and other scenarios. With the improvement of devices, people can collect multi-sensor/multimodal data from a variety of sensors such as Lidar and cameras. In order to make full use of various information advantages and improve the performance of object detection, we proposed a Complex-Retina network, a convolution neural network for 3D object detection based on multi-sensor data fusion. Firstly, a unified architecture with two feature extraction networks was designed, and the feature extraction of point clouds and images from different sensors realized synchronously. Then, we set a series of 3D anchors and projected them to the feature maps, which were cropped into 2D anchors with the same size and fused together. Finally, the object classification and 3D bounding box regression were carried out on the multipath of fully connected layers. The proposed network is a one-stage convolution neural network, which achieves the balance between the accuracy and speed of object detection. The experiments on KITTI datasets show that the proposed network is superior to the contrast algorithms in average precision (AP) and time consumption, which shows the effectiveness of the proposed network.

## 1. Introduction

In the field of object detection, the existing methods mostly use a camera with CCD or CMOS sensors to acquire images for two-dimensional (2D) object detection [1,2]. This part of the research has a long history. With the rapid development of deep learning, these methods based on deep neural networks have made great progress both in detection accuracy and real-time capability and have been achieving important applications in many fields such as medicine, commerce and engineering [3,4,5]. However, in many practical applications such as robotics, automatic loading and autonomous driving, more attention is paid to the three-dimensional (3D) position information of objects. Therefore, it is necessary to develop 3D object detection methods [6]. Lidar has the advantages of not being affected by external illumination and having a high accuracy, but its resolution is far lower than the camera. Fusing the data from Lidar and cameras for 3D object detection can achieve the effect of complementary advantages, so it has attracted the attention of researchers.

At present, 2D object detection methods in the field of imaging mainly focus on deep learning and have achieved desirable results. Generally speaking, those methods can be divided into two-stage and one-stage networks. Since R-CNN [7] first introduced convolutional neural networks into object detection, a two-stage detection framework has been established, and with the proposals of Fast R-CNN [8], Faster-RCNN [9], RFCN [10] and other networks, this series of networks has been continuously improved. Those networks have two stages: in the first stage, a Region Proposal Network (RPN) is trained to extract a certain number of candidate bounding boxes, and then in the second stage, classification and regression of candidate bounding boxes are carried out more carefully to increase detection precision. In addition, new ideas about two-stage networks have been generated: Cascaded RCNN [11] cascaded several detection networks based on positive and negative samples determined by different IoU (Intersection over Union) thresholds to continuously optimize the prediction results. In reference [12], Relation Networks proposes an object relation module to describe the relationship between objects. It optimizes the detection effect by utilizing the relationship between objects in an image, or image context. SNIP [13] proposes a new training model: when the gradient returns, only the gradient of ROI (Region of Interest) corresponding to the size of training data used by the pre-training model is retrieved, which reduces the domain-shift between the ImageNet [14] dataset and the COCO [1] dataset. Generally speaking, two-stage networks have attracted wide attention from all walks of life. In contrast, one-stage networks do not need the rough extraction of RPN, but directly complete the classification and regression of the object bounding boxes from the image, so the one-stage networks often have higher real-time performance. Since YOLO [15] and SSD [16] networks were proposed, this framework has also received attention. After that, improved versions such as YOLOv2 [17], YOLOv3 [18], DSSD [19] appeared, and performance continued to improve. In reference [20], Detection with Enriched Semantics (DES) is mainly based on SSD to improve the semantic information of feature maps of each level by introducing a segmentation module and global activation module, thus improving the detection effect of small objects. RetinaNet [21] proposes a new loss function, Focal Loss, which greatly improves the detection performance of one-stage networks. Scale-Transferrable Detection Network (STDN) [22] introduces a scale-transfer layer to generate feature maps of a large size without increasing the number of parameters and computation. RefineDet [23] combines an SSD algorithm with an RPN network and Feature Pyramid Network (FPN) [24] algorithm to improve the detection effect while maintaining the efficiency of SSD.

Similarly, 3D point cloud object detection can be divided into two-stage and one-stage network structures. At present, the most accurate detection methods are based on two-stage architecture, such as MV3D [25], AVOD [26] and VoxelNet [27]. MV3D was the first network to extend RPN to 3D point clouds. It designed unified network architecture to fuse images and point clouds and the main idea was to extract some features to express the point cloud into the form of front view and bird’s-eye view (BEV) with 3D information, achieving a good detection effect. AVOD improved MV3D with some corrections, in which only point cloud BEV and image data were used for input and the backbone network was changed to FPN architecture, which improves the feature map size and is more conducive to small object detection, thus improving the detection precision. VoxelNet first divided the point cloud into voxels and extracted the features of the voxels using the cascaded VFE layer, and then used the RPN network for object detection. F-PointNet [28] first uses image to detect, and then projects the 2D detection results into point cloud to form frustum, which is segmented by PointNet [29]. Then the 3D bounding boxes of the object are calculated. This network has achieved better results in the detection of bicycles and pedestrians, but it cannot run end-to-end. Because all the above methods need RPN network to select candidate bounding boxes with high probability of object from a large number of unknown boxes, and then conduct more detailed classification and regression, it is difficult to meet the actual real-time needs.

Since the one-stage detector has the advantage of fast speed, in order to solve the above problem, both Complex-YOLO [30] and YOLO3D [31] networks extend YOLO to 3D point cloud object detection, which achieves good real-time performance, but the AP is low. PIXOR [32] is proposed also as a one-stage network structure. This network does not need RPN operation. It directly performs box regression and classification operations from feature maps, which meets the real-time requirements. However, the network can only detect 2D bounding boxes in the BEV of point clouds but cannot output 3D bounding boxes. In addition, the network does not use anchors, which is also a reason for poor performance.

In this paper, a one-stage convolutional neural network, Complex-Retina, is proposed for 3D object detection. We divide the network into two parts: backbone network and functional network. The former performs feature extraction and effective fusion for multi-sensor data i.e., point clouds and images, and the latter is used for the classification and regression of 3D bounding boxes. We draw lessons from RetinaNet, which is a one-stage 2D detection network and has achieved good results with images, and we also modify the network to some extent. The backbone network is combined with two paths of FPN for the feature extraction of point clouds and images. For the sake of facilitating the fusion of multi-sensor data, we set a series of 3D anchors and implemented ROI pooling and fusion on the feature maps of those two kinds of data. In addition, a functional network adapted to the 3D data was designed to output 3D bounding boxes and category information. These modifications extended the RetinaNet to a 3D multi-sensor fusion detector and improved the detection performance of the one-stage 3D object detection network with comparable capability to the two-stage networks while maintaining the speed advantage. The experimental results on the KITTI dataset [33] show the effectiveness of the proposed network. The main contributions of the proposed network include the following two points: (1) through a series of improvement methods, RetinaNet is extended from 2D to 3D detection; and (2) the proposed one-stage 3D detection network can balance the detection speed and precision, and has a good application prospect.

## 2. Review of RetinaNet

RetinaNet [21] is essentially composed of FPN structure and Focal Loss. The design idea is that the backbone chooses effective feature extraction networks such as VGG [34] and ResNet [35]. Then, FPN is used to obtain feature maps with stronger expression and multi-scale object information. Finally, a two-path FCN subnetwork is used on the feature maps to complete the classification and regression tasks for the bounding boxes. 

Anchor information: 12 anchors are set for each pixel, ranging in area from 322 to 5122.There are three different ratios of length to width {1:2,1:1,2:1} on each level of the feature pyramid from P3 to P7. For denser scale coverage, three different size weights {20, 21/3, 22/3} are added to each level anchor set. Finally, the network allocates a vector with length (K+4) to each anchor to carry classified information and bounding box regression information.

Training and deployment of the model: the top 1,000 bounding boxes with the highest probability were selected at each FPN level, and then all bounding boxes at all levels were summarized and filtered using an NMS with a threshold of 0.5. The loss function is composed of L1 Loss and Focal Loss. The former was used to measure the regression error, whereas the latter was used to measure the classification error, and it can well solve the extreme imbalance between positive and negative samples.

## 3. Complex-Retina

In order to extend RetinaNet to the 3D domain, we have mainly made the following inheritance and improvement:

- The backbone network has two paths of sub-network for the feature extraction of point clouds and images. For each path, the first four layers of VGG network are selected and FPN structure is adopted, which is similar with RetinaNet.
- There are differences in anchor settings. Every anchor is represented by the 6-dimensional array of length, width, height and center point coordinates, and the clustering method is used to set the size of anchors.
- There are also differences in ROI Pooling. By projection method, we used 3D anchors to perform ROI Pooling and fusion on the feature maps of point clouds and images. In addition, unlike the multi-output strategy of RetinaNet, our backbone network only performs ROI Pooling and fusion on the feature map of P7 layer of the FPN.
- In the design of loss function: Focal Loss is also used to measure the classification error and L1 function is used to measure the regression error of bounding box. In addition, orientation error is added, which is very important for 3D object detection.

**Figure 1:**
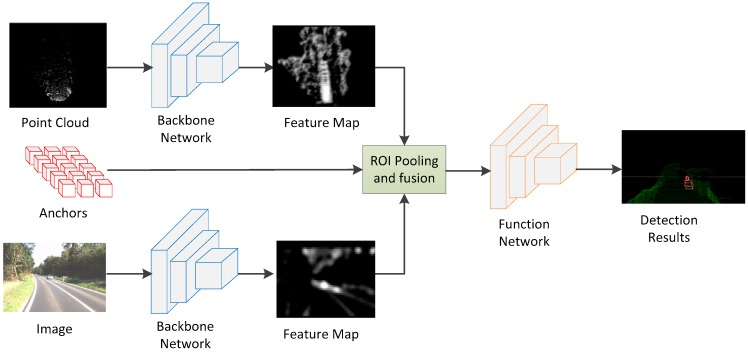
The architecture of our proposed Complex-Retina network. It can be divided into the backbone network and functional network. The backbone network is used to extract the feature maps of point cloud and images, and then the feature maps are sent to the functional network after ROI Pooling and fusion, which outputs the classification and regression information of the objects.

The structure of 3D object detection network designed in this paper is shown in Figure 1, which is mainly divided into the backbone network and functional network. The backbone network is used to extract the feature maps of point cloud and images, and then the feature maps are sent to the functional network after ROI Pooling and fusion, which outputs the classification and regression information of the objects.

### 3.1. Input Data

We adopted a KITTI dataset for network training and validation. The dataset is jointly published by Karlsruhe Institute of Technology and Toyota American Institute of Technology. It uses cameras and Lidar sensors. This dataset contained a large number of images and point cloud data with annotated information, and they needed to be preprocessed before input to the proposed network.

The images in the KITTI dataset were collected by two color cameras and two gray-scale cameras. We only used the image captured by the left color camera and each of the input images was cut into 360 × 1200.

The point clouds in KITTI datasets were collected by vehicle-mounted Velodyne 64-line LIDAR, which provided accurate object location information. The dataset took the forward direction of the car as the X coordinate, the upward direction as the Y coordinate, and determined the Z coordinate according to the right-hand rule. In order to process point clouds using deep neural network, this paper presents point clouds as BEV, which has the following advantages: first, less occlusion. For vehicle scenarios, BEV had less occlusion than the front view. Second, it can be processed by a mature 2D object detection network as backbone. In order to transform point clouds into BEV, this paper used 0.1 m × 0.1 m interval to grid point clouds on the X and Z axes, whereas on the Y axis, the point clouds were evenly divided into five layers in the range of [0,2.5] m, so the whole point clouds were divided into several cell units. The elevation information of the highest point in each cell was preserved, and the five layers made up the five channels of the BEV. In addition, the density information of point cloud was recorded in the sixth channel, and the calculation method was as follows: $\min\left(1.0,\frac{\log\left(N+1\right)}{\log16}\right)$ [25]. Since only the forward [−40,40] m × [0,70] m point clouds were retained, the final size of the input point clouds BEV was 700 × 800 and the number of channels was six. Before input to the backbone network, the BEVs were padding to 704 × 800 to facilitate the three layers of Maxpooling processing in the backbone network.

### 3.2. Backbone Network

**Figure 2:**
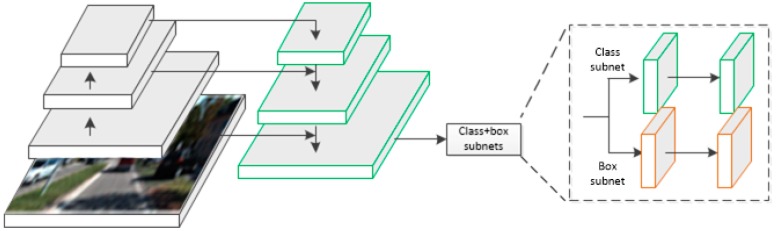
The structure of Backbone network. Our backbone uses the FPN architecture but only outputs the last layer of feature map to the functional network to estimate the detection results.

In this paper, the first four layers of VGG-16 were used in the design of the backbone network, with the channel numbers of all the layers were modified, and the specific parameters are shown in Table 1. The backbone network was used for feature extraction. By stacking convolution layers and pooling layers one by one, the channel number of each layer was continuously increased and the size of feature map was reduced. Usually, low-level feature maps have high resolution and low expressive ability (i.e., low-level geometric information). On the contrary, high-level feature maps have low resolution and high expressive ability (i.e., high-level semantic information). Most networks output the last layer of feature maps for subsequent classification and detection tasks, which often leads to small objects occupying few pixels, which is not conducive to detection. The FPN structure [24] can effectively solve this problem. As shown in Figure 2, the backbone network treated the normal top-down down-sampling process as an encoder and added a bottom-up decoder. The output feature map of the conv4 layer of the encoder was used as the input of the decoder, and then it was fused with conv3. Firstly, the conv4 layer feature map was convtransposed to conv_trans3 by 3 × 3 convolution kernel. In this process, the size of the conv4 feature map was enlarged and the channels were reduced, which was consistent with the conv3 feature map to achieve the effect of up-sampling. Then the conv_trans3 feature map was combined with the conv3 feature map by concatation, and the pyramid3 layer fused the concat_3 feature map by convolution.

By repeating the above operations layer by layer, the final pyramid1 feature map had the same size as the input data, which can facilitate ROI pooling. At the same time, the feature map had both high resolution and expressive ability, which was very helpful for the detection task.

In terms of output strategy, this paper is different from the multi-scale output mode of RetinaNet. RetinaNet will perform ROI pooling operations at each level of the feature pyramid, and then input proposals of various scales into the functional network for filtering. This method improves the detection effect by expanding the redundancy, but at the same time, it also increases the amount of computation. In this paper, the backbone network only outputs the last feature map of the FPN to the functional network to estimate the results, which reduces the computational complexity compared with the multi-scale strategy.

### 3.3. ROI Pooling and Multi-Sensor Fusion

In order to effectively carry out the regression of bounding boxes, we did not output directly from the feature maps. Instead, a certain number of anchors were set up so that we could simply predict the deviation of the anchors. For the feature maps output from backbone network, the 3D anchors were projected into them first, then the feature maps were cropped according to the projection results, and a large number of feature map 2D anchors with the same size were obtained. The ROI pooling operation on both BEV and image feature maps was completed. Finally, the feature fusion of the multi-sensor feature maps was carried out with element-wise mean to obtain 2D fusion anchors.

Anchors refer to the pre-set inaccurate bounding boxes arranged densely according to certain rules. In addition, these kinds of boxes usually cannot surround the target well, so they need to use the neural network for regression. That is to say, anchors can help network output bounding boxes. In the following Section 3.4, those 2D fusion anchors are sent to the functional network for final classification and regression. Unlike our method, the PIXOR [32] network does not set anchors, but directly regresses the bounding boxes from the feature maps, so it is difficult to achieve better results. In fact, the method of setting anchors has been used in many networks such as SSD [16] and YOLOv2 [17]. YOLOv2 sets five anchors for each grid unit to adapt to different distances and sizes of objects. However, we conducted the experiments with cars on the KITTI dataset as objects, considering the data collected by mobile LIDAR, and the size of vehicles will not vary too much, so in order to save unnecessary computational complexity, only two sizes of anchors were set up in this paper.

#### 3.3.1. D Anchor Setting

The commonly used encoding method of 3D bounding boxes is represented by 24-dimensional data of eight corners, but it cannot guarantee the alignment of the positions of those corners. In this paper, an anchor is represented by six parameters of center coordinate (x, y, z) and length, width and height (l, w, h). Firstly, in the BEV of point cloud, the x and y values were obtained by uniform sampling at 0.5 m intervals, whereas the z value was determined by the height of the sensor from the ground. Then, for the size of the anchors, they can be determined by clustering the label information of the vehicle object in the dataset as follows: [3.513, 1.581, 1.511] and [4.234, 1.653, 1.546]. Because of the sparsity of point clouds, some 3D anchors did not contain point clouds and needed to be removed. Integral image is widely used in image processing, and the value of each position in the integral image is the sum of all the pixels in the upper left corner of the position in the original image. Similarly, we can calculate the sum of point clouds in the 3D anchors, thus removing the anchors with the result of zero.

#### 3.3.2. ROI Pooling and Fusion with Anchors

In order to perform feature fusion, it is necessary to conduct ROI pooling on the feature maps. So, we projected the 3D anchors onto the feature maps of BEV and images, and then, conducted cropping and resizing on them. Since the 3D anchors were set on the BEV, the occlusion was not considered in the projection process of BEV. For the 3D anchor $\left(x_{t},y_{t},z_{t},l,w,h\right)$, the upper left corner and the lower right corner of the projection region on the BEV can be expressed as $\left(x_{l,\min},z_{l,\min}\right)$ and $\left(x_{l,\max},z_{l,\max}\right)$:

(1) $$\left\{ \begin{array}{l} x_{l,\min}=x_{t}-\frac{l}{2}\\ x_{l,\max}=x_{t}+\frac{l}{2}\\ z_{l,\min}=z_{t}-\frac{h}{2}\\ z_{l,\max}=z_{t}+\frac{h}{2} \end{array}\right.$$

**Figure 3:**
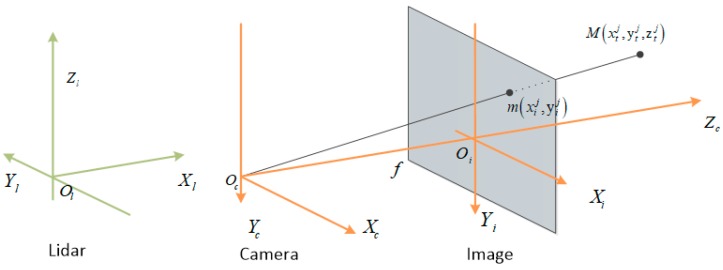
Multi-sensor coordinates. For the vehicle-borne multi-sensor data acquisition system, the Lidar coordinate system $O_{l}-X_{l}Y_{l}Z_{l}$, image coordinate system $O_{i}-X_{i}Y_{i}Z_{i}$ and camera coordinate system are different, they can be transformed to each other when necessary.

For the 3D anchor projection to the image feature maps, the calculation process was more complex. Because the KITTI data acquisition system has many coordinate systems as shown in Figure 3 [33], in which point cloud and 3D anchors are represented in the Lidar coordinate system, $O_{l}-X_{l}Y_{l}Z_{l}$, while images are in the image coordinate system $O_{i}-X_{i}Y_{i}Z_{i}$, it is necessary to transform the 3D anchors parameters into the image coordinate system.

Firstly, it is easy to calculate the coordinates of 8 vertices $\left(x_{t}^{j},y_{t}^{j},z_{t}^{j}\right),j=1\cdots 8$ of the anchor according to its center point, length, width and height.

Then the vertex coordinates were transformed into image coordinates. For the transformation from Lidar coordinates to camera coordinates, it was necessary to multiply the corresponding transformation matrix $T_{lid}^{cam}$. If a vertex in an anchor is set as $M\left(x_{t}^{j},y_{t}^{j},z_{t}^{j}\right)$, the transformation into camera coordinates can be expressed as:

(2) $$(x_{c}^{j},y_{c}^{j},z_{c}^{j})=T_{lid}^{cam}\cdot \left(x_{t}^{j},y_{t}^{j},z_{t}^{j}\right)$$

where, the transformation matrix $T_{lid}^{cam}$ is provided by the dataset. According to the imaging projection relationship, the point *m* in the camera coordinate system can be converted to the image coordinate system as $m\left(x_{i}^{j},y_{i}^{j}\right)$:

(3) $$\{\begin{array}{c} \frac{x_{i}^{j}}{f}=\frac{x_{c}^{j}}{z_{c}^{j}}\\ \frac{y_{i}^{j}}{f}=\frac{y_{c}^{j}}{z_{c}^{j}} \end{array}$$

Its matrix form is:

(4) $$z_{c}^{j}\left[\begin{array}{c} x_{i}^{j}\\ y_{i}^{j}\\ 1 \end{array}\right]=\left[\begin{array}{c} \begin{array}{cccc} f & 0 & 0 & 0\\ 0 & f & 0 & 0\\ 0 & 0 & 1 & 0 \end{array} \end{array}\right]\left[\begin{array}{c} x_{c}^{j}\\ y_{c}^{j}\\ z_{c}^{j}\\ 1 \end{array}\right]$$

where, *f* denotes the focal length of the camera. Equations (2) and (4) are used to transform the 3D anchors from the Lidar coordinate system to the image coordinate system to obtain $\left(x_{i}^{j},y_{i}^{j}\right),j=1\cdots 8$. Considering the occlusion effect, the vertices of the projection area of the 3D anchors can be expressed as follows:

(5) $$\{\begin{array}{cc} \begin{array}{l} x_{i,\min}=\min\left(x_{i}^{j}\right)\\ x_{i,\max}=\max\left(x_{i}^{j}\right)\\ y_{i,\min}=\min\left(y_{i}^{j}\right)\\ y_{i,\max}=\max\left(y_{i}^{j}\right) \end{array} & ,j=1\cdots 8 \end{array}$$

According to the parameters of the projection area of the 3D anchors, the feature map was cropped and resized to 7 × 7, and the feature map anchors were in the same size. In this way, the 2D feature map anchors can be corresponded with the 3D anchors. And the feature fusion of the multi-sensor feature maps is completed with an element-wise mean.

During the training phase, those feature fusion anchors were marked positive and negative by calculating the IoU between the anchors and ground truth bounding boxes, the anchors were recorded as positive samples when the IoU was greater than the threshold, and vice versa.

### 3.4. Functional Network

**Figure 4:**
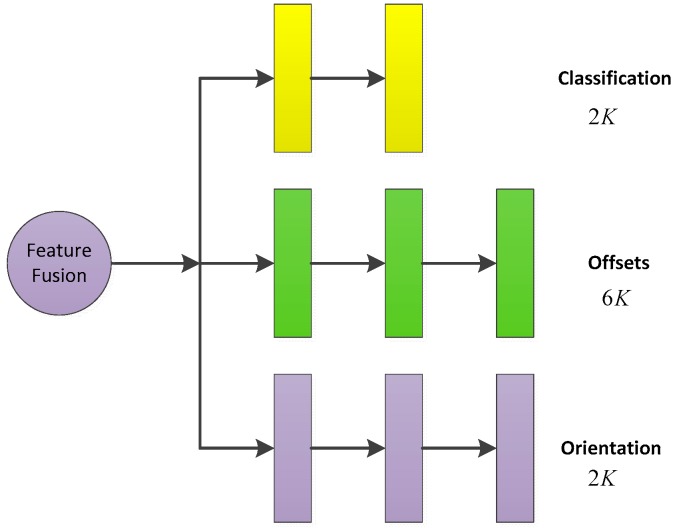
The output of fully connected layers. When the feature fusion anchors with the total number of K, the output dimensions of classification FC network is 2K; the output dimensions of bounding box regression FC network is 6K; the output dimensions of orientation regression FC network is 2K.

The samples of feature fusion anchors were input to the functional network for classification judgment and regression of offset. The final prediction bounding boxes were based on the 3D anchors with corresponding offsets. Compared with the traditional method of direct regression of bounding boxes, this method not only reduced the difficulty of regression, but also located more accurately. As shown in Figure 4, the functional network consisted of three parallel multi-path fully connected layers. The three fully connected networks performed three tasks: classification, bounding box regression and orientation regression.

#### 3.4.1. The Output Size

For the feature fusion anchors with the total number of K, the classification information included the object and background, and the total output dimension of the classification network was 2K. For the bounding box regression network, in order to express the offset values of the center coordinates and the length, width and height between the anchors and the ground truth bounding boxes, the regression result was set as $\left(\Delta x_{t},\Delta y_{t},\Delta z_{t},\Delta l,\Delta w,\Delta h\right)$, and the output dimension was 6K. For the regression of the orientation of the bounding box, this paper adopted the method of calculating the angle vector $\left(x_{or},y_{or}\right)=\left(\cos\left(\theta \right),\sin\left(\theta \right)\right)$ of the projection of the bounding box in the BEV, so the output dimension was 2K.

#### 3.4.2. Loss Function

In this paper, a multi-task loss function was designed. Two smoothing L1 functions were used to measure the errors of regression of bounding box and orientation, and the Focal Loss function [21] was used to measure the classification errors.

(6) $$L\left(\left\{p_{i}\right\},\left\{t_{i}\right\}\right)=\frac{1}{N_{cls}}\sum_{i}L_{cls}\left(p_{i},p_{i}^{*}\right)+\lambda \frac{1}{N_{reg}}\sum_{i}p_{i}^{*}L_{reg}\left(t_{i},t_{i}^{*}\right)+\lambda \frac{1}{N_{ang}}\sum_{i}p_{i}^{*}L_{ang}\left(t_{i},t_{i}^{*}\right)$$

where, symbol *i* represents the index of the anchor, and symbol $p_{i}$ represents the probability value of the predicted object. $p_{i}^{*}$ means the annotation information, and positive and negative samples are labeled as 1 and 0 respectively. $t_{i}$ represents the predicted result of the bounding box, and $t_{i}^{*}$ is the label of the positive example. 

The first item on the right side of the equal sign is the loss value between the predicted category and the ground truth, and the second item indicates the loss value between the estimated bounding box and the ground truth, where $p_{i}^{*}L_{reg}$ indicating that the item is only related to the positive samples, and for those estimated bounding boxes with IoU less than 0.65 are recorded as negative samples, which are labeled as 0. The third item shows the deviation between the predicted and ground truth of the orientation angle. In the formula, $N_{cls}$, $N_{reg}$ and $N_{ang}$ are used to normalize the above three items (respectively 5, 7 and 1), whereas the hyper-parameter λ is used to balance the weights among the items.

The Focus Loss was used as our classification loss function. Because the background was the majority of samples and object bounding boxes were only a small part of samples, there was a problem of class imbalance and the network spent a lot of computing resources on the non-object samples, which greatly reduced the training effect. Focal Loss reduced its impact by reducing the weight of large-number samples, which effectively solved the problem. The parameters α and β in Focus Loss were set to 0.25 and 2, respectively.

## 4. Experiment Results and Analysis

Dataset introduction: In this paper, the network was trained and tested on the KITTI dataset, in which the samples were divided into three levels: easy, medium and difficult, according to the bounding box height, occlusion level and truncation. Because the annotation information of the test set was not openly available, this paper divided 3769 samples from the training set with 7481 samples into the validation sets, which were only used in the test phrase.

Sample selection: This paper adopted the end-to-end method to train the network. Each time the numbers of point cloud file and image were input into the network both were set as one. After feature extraction of the backbone network, ROI Pooling and feature fusion were carried out to produce the feature map anchors for the functional network. In this stage, the upper limit of the number of batch training was set as 16,384. When the number of feature map anchors in this paper was larger than this upper limit, down-sampling processing was needed. Since the number of positive anchors was usually few, only negative anchors were down-sampled.

Training details: the ADAM function was used to carry out 150,000 iterations in the network. The initial learning rate was 0.0001, which was attenuated exponentially with a coefficient of 0.1 and an attenuation step of 100,000. In order to prevent overfitting during the training stage, the dropout of each layer in the backbone network was set to be 0.9, and the dropout of the fully connected layer was set to be 0.5. In addition, batch normalization was used for all the fully connected layers. Finally, when there was a corresponding relationship between multiple anchors and one ground truth bounding box, the non-maximum suppression (NMS) method was used to eliminate the redundant anchors, and the threshold was set to 0.01. The GPU type used in the test phase was TITAN Xp.

### 4.1. Experiment 1: Feature Extraction Performance

**Figure 5:**
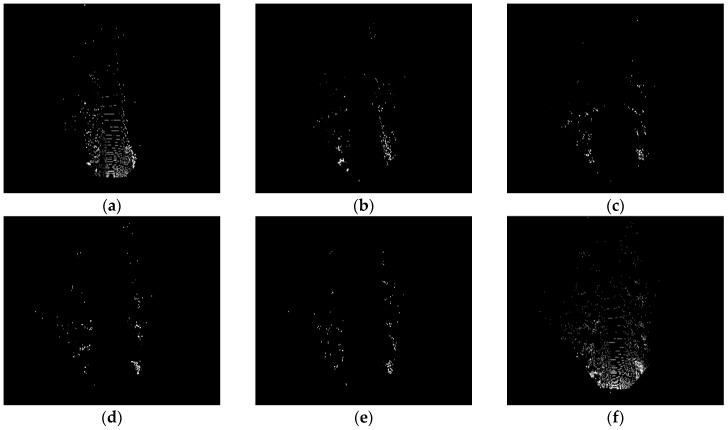
Input point cloud BEV. According to the method described in Section 3.1, we obtain the BEV of point cloud as input for the network. (**a**–**e**): Five Elevation diagrams; (**f**): Density map.

**Figure 6:**
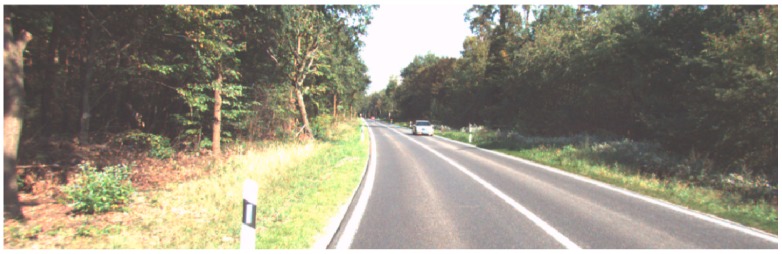
Corresponding image input.

**Figure 7:**
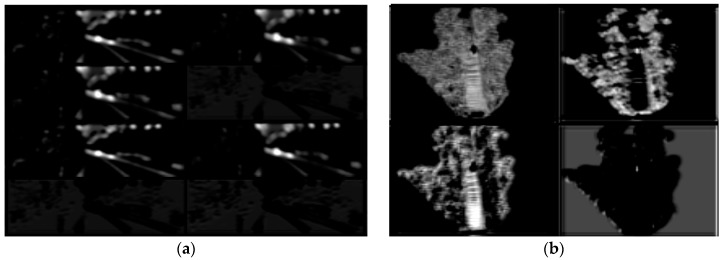
The output feature maps from con4_3 layer of backbone network: (**a**) the feature maps of image; (**b**) the feature maps of BEV.

Figure 5 shows the input point cloud BEV, where Figure 5a–e are the elevation diagrams, Figure 5f is the density map, and Figure 6 is the corresponding picture, in the center of which a vehicle can be clearly seen. In order to test the feature extraction performance of backbone network, we input the BEV and image into the trained backbone network and visualize the feature maps of them in the Figure 7.

Figure 7 shows part of the feature maps from the last layer (i.e., con4_3) of the backbone network when the input data were processed. Eight channels of the image feature map (Figure 7a) and four channels of the BEV feature map (Figure 7b) were selected for visualization. It can be seen from the Figure 7 that part of the feature maps has a relatively obvious response to the position of the object i.e., vehicle, indicating that the backbone network adopted in this paper can effectively extract the feature maps of the input data and lay a good foundation for detection.

**Figure 8:**
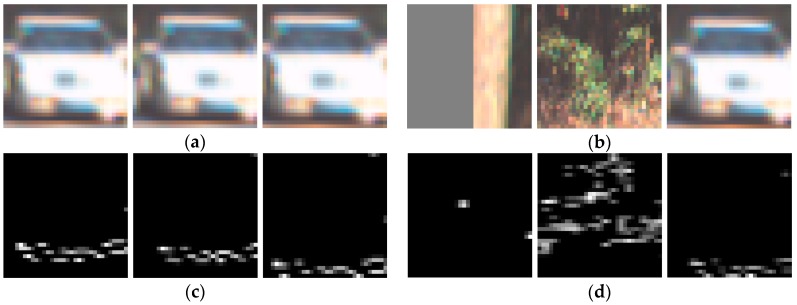
The features maps cropped using 3D anchors: (**a**,**b**) the feature maps of image; (**c**,**d**) the feature maps of BEV.

In order to perform ROI pooling and feature fusion, we needed to project the 3D anchors into the point cloud BEV and image feature maps and crop the feature map into 2D anchors. Figure 8a,b show six image feature map anchors (size of 7 × 7) after cropping, and four of which contain the object of vehicle. Figure 8c,d are the cropped samples of corresponding BEV feature maps. It can be seen that there were great differences between image feature maps and BEV feature maps, and they are highly complementary.

### 4.2. Experiment 2: 3D Object Detection Performance

The results in the validation set are shown in Table 2. The 3D detection results can be measured by average precision (AP). The threshold was set to 0.7. When the IoU value was greater than the threshold, the detection was successful.

As can be seen from Table 2, for the three tasks of the dataset, namely Easy, Moderate and Hard, the proposed method surpassed the other methods in detection performance. The network BirdNet [36] and RT3D [37] only used the point cloud data of LIDAR, since the resolution of the point cloud data was much lower than that of the image, it was not conducive to the detection of small objects, so the AP was relatively low. Complexer-YOLO [30] characterizes the height, intensity and density of point clouds as RGB-map input, uses 18 convolution layers and 5 maxpooling layers for feature extraction, then uses YOLO-like loss function in the design of loss function. However, due to the unbalanced classification of object and background, too many negative samples are not conducive to the further improvement of network performance. The A3DODWTDA [38] network uses images to detect two-dimensional objects first, then projects them into 3D space to obtain the possible regions of objects and segment the object point clouds. Finally, the 3D detection results of objects were obtained by regression. Because the method divided images and point clouds into two steps separately, it did not achieve effective fusion effect, so the detection effect was not good. The MV3D [25] network effectively fused the features of image and point cloud, and the detection performance surpasses the above algorithms. In terms of timeliness, since the network adopts two-stage network design, it runs slowly. The network proposed in this paper achieved the fusion of multi-sensor data and good detection results. Moreover, the problem of class imbalance was solved, so the detection accuracy was higher than that of the above-mentioned network, and because of the one-stage network design, the running time was also better than that of MV3D.

### 4.3. Experiment 3: BEV Object Detection Performance

We also provide experimental results on BEV with the KITTI dataset, setting the IoU of BEV to 0.7. As shown in Table 3, the performance of the A3DODWTDA and MV3D networks that combine images with point clouds exceeds the Complexer-YOLO that uses only point clouds. The PIXOR network is the best one-stage point cloud BEV detection network at present. Its design is better than Complexer-YOLO network, for example, the introduction of FPN in the backbone network is beneficial for detection and PIXOR also refers to Focal Loss in the loss function. Therefore, the final detection result is even better than the two-stage network MV3D. However, PIXOR does not use anchors, and does not design 3D regression operations, resulting in the inability to obtain 3D information. The proposed network in this paper has improved this by introducing anchors and 3D information fusion, and our BEV detection results surpass PIXOR.

### 4.4. Experiment 4: Accuracy-Recall Curve

**Figure 9:**
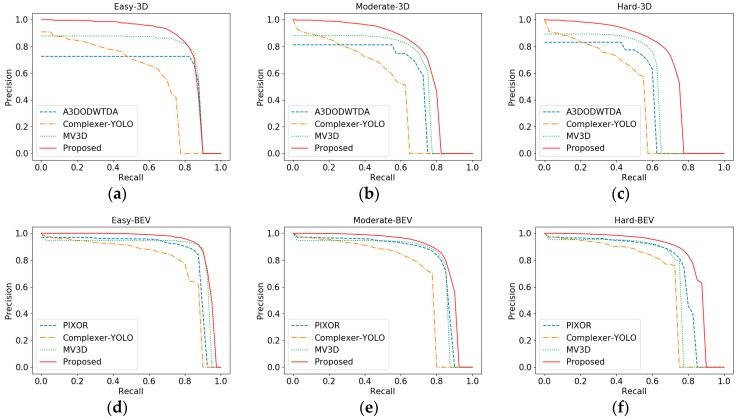
Accuracy-recall curve: (**a**) Easy task in 3D detection; (**b**) Moderate task in 3D detection; (**c**) Hard task in 3D detection; (**d**) Easy task in BEV detection; (**e**) Moderate task in BEV detection; (**f**) Hard task in BEV detection.

Figure 9a–c are the accuracy-recall curves of 3D detection, which correspond to three difficulties: Easy, Medium and Difficult. It can be seen from the figures that the performance of Complexer-YOLO as one-stage network was obviously lower among the three tasks, whereas the proposed network in all the three tasks was significantly better than the other three networks, which shows that the proposed network has certain advantages in the detection of different cases. For the “Easy” task, A3DODWTDA and MV3D can maintain longer and higher precision too, since both networks are two-stage networks, with the improvement of recall, the number of proposals also increases, which can give full play to the screening function of RPN subnetwork. However, the proposed network is superior to the two networks in precision retention.

Similarly, Figure 9d–f are the BEV test results. Here we focus on comparing with the best one-stage BEV detection network PIXOR. It can be seen that in the three difficulties, the proposed network is obviously better than PIXOR. Moreover, it is worth noting that in terms of BEV detection, the proposed network also has some advantages over MV3D, which further indicates the effectiveness of the proposed network.

**Figure 10:**
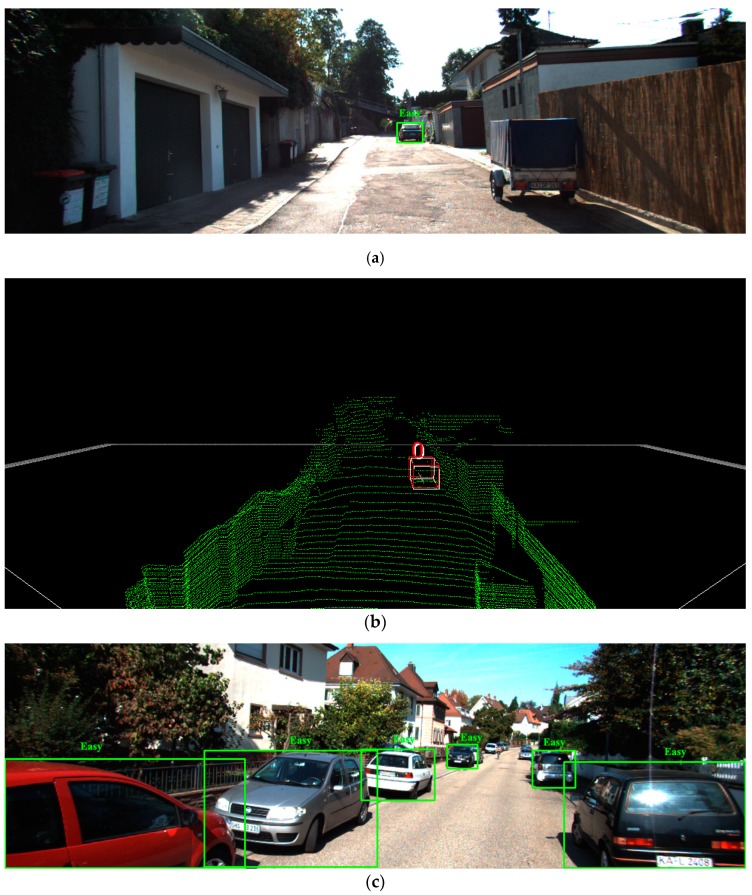

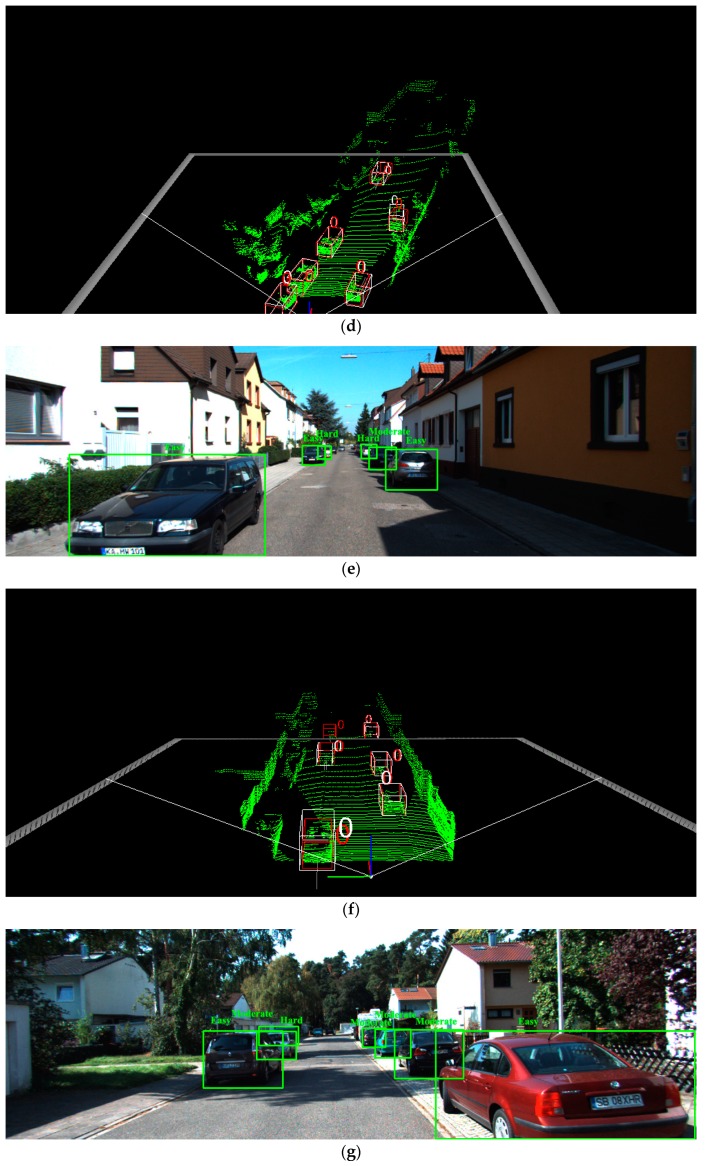

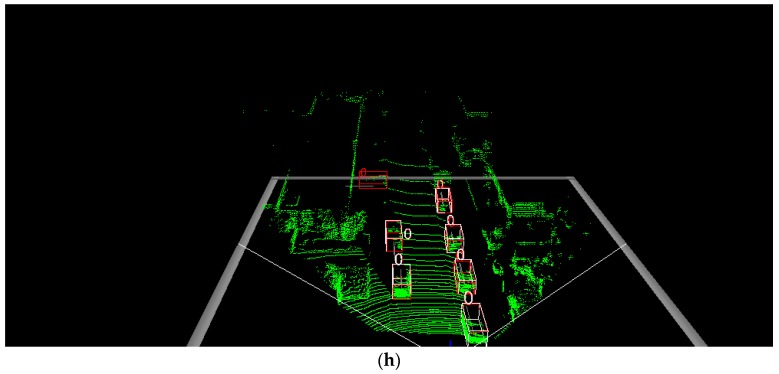
Detection results of two samples for the *Val* dataset: (**a**) image 000002; (**b**) point cloud 000002; (**c**) image 000008; (**d**) point cloud 000008; (**e**) image 000039; (**f**) point cloud 000039; (**g**) image 004118; (**h**) point cloud 004118.

Figure 10 shows some visualization results of the detection. Figure 10a,c,e,g correspond to four images whose index numbers are 000002, 000008, 000039 and 004118, respectively. The difficulty of object detection in each image is marked. We can see that the bigger the occlusion part is, the more difficult the detection is. Figure 10b,d,f,h show the 3D detection results corresponding to the four samples mentioned above. In the figures, the red 3D bounding boxes are the ground truth and the white ones are the estimated results. From the figures, it can be seen that the proposed network can achieve satisfactory detection results on objects labeled as Easy and Moderate. But for the objects labeled as Hard, there are several cases of missed detection. For example, in Figure 10f,h, we can see that the innermost object has not been successfully detected because of the serious occlusion and very few point clouds can be received from these objects. The detection of Hard targets will be the focus of our next stage of research.

## 5. Conclusions

The application of multiple sensors makes it possible for people to carry out more accurate object detection. In this paper, a convolutional neural network for 3D object detection was proposed. By improving the existing RetinaNet, a unified architecture for 3D object detection was designed. The network can be divided into two parts: the backbone network and the functional network. Firstly, the backbone network performs feature extraction on point cloud BEV and images. Then, ROI pooling and feature fusion are performed on the feature map, and the feature map is cropped and resized by projecting the 3D anchors into the feature map of BEV and image respectively. Finally, multi-path fully connected layers are used to conduct the object classification and the regression of 3D bounding boxes. In addition, the designed network is a one-stage convolutional neural network without the use of RPN and it achieves the balance between object detection precision and detection speed. The validation experiments on the KITTI dataset show that the proposed network is superior to the baseline in terms of AP and time consumption.

In the data preprocessing stage, we set the parameters of 3D anchors according to the distribution characteristics of the KITTI dataset, so it is necessary to adjust the anchors when applying the network to other datasets, which is not conducive to the portability and universality of the network. This defect will be the focus of our follow-up study.

## Figures and Tables

**Table 1:** The parameters of Backbone network layers. Since the network has two paths to process point cloud bird’s eye view (BEV) and images separately, their parameters in each layer are different.

Input	BEV		Image	
Part	Layers	Output Size	Layers	Output Size
**Encoder**	conv1_1	704 × 800 × 32	conv1_1	360 × 1200 × 32
	conv1_2		conv1_2	
	maxpooling	352 × 400 × 32	maxpooling	180 × 600 × 32
	conv2_1	352 × 400 × 64	conv2_1	180 × 600 × 64
	conv2_2		conv2_2	
	maxpooling	176 × 200 × 64	maxpooling	90 × 300 × 64
	conv3_1	176 × 200 × 128	conv3_1	90 × 300 × 128
	conv3_2		conv3_2	
	conv3_3		conv3_3	
	maxpooling	88 × 100 × 128	maxpooling	90 × 300 × 128
	conv4_1	88 × 100 × 256	conv4_1	45 × 150 × 256
	conv4_2		conv4_2	
	conv4_3		conv4_3	
**Decoder**	conv_trans3	176 × 200 × 128	conv_trans3	90 × 300 × 128
	concat3	176 × 200 × 256	concat3	90 × 300 × 256
	pyramid3	176 × 200 × 64	pyramid3	90 × 300 × 64
	conv_trans2	352 × 400 × 64	conv_trans2	180 × 600 × 64
	concat2	352 × 400 × 128	concat2	180 × 600 × 128
	pyramid2	352 × 400 × 32	pyramid2	180 × 600 × 32
	conv_trans1	704 × 800 × 32	conv_trans1	360 × 1200 × 32
	concat1	704 × 800 × 64	concat1	360 × 1200 × 64
	pyramid1	704 × 800 × 32	pyramid1	360 × 1200 × 32

**Table 2:** Detection Results of Different Methods.

Method	Structure	Input	Runtime/s	Easy/%	Moderate/%	Hard/%
BirdNet [36]	2-stage	Lidar	0.11	14.75	13.44	12.04
RT3D [37]	2-stage	Lidar	0.09	23.49	21.27	19.81
Complexer-YOLO [30]	1-stage	Lidar	0.06	55.63	49.44	44.13
A3DODWTDA [38]	2-stage	Lidar + img	0.08	59.35	56.81	50.51
MV3D [25]	2-stage	Lidar + img	0.36	71.09	62.35	55.12
Proposed	1-stage	Lidar + img	0.09	78.62	72.77	67.21

**Table 3:** Detection Results of Different Methods in BEV.

Method	Structure	Input	Runtime/s	Easy/%	Moderate/%	Hard/%
Complexer-YOLO [30]	1-stage	Lidar	0.06	74.23	66.07	65.70
A3DODWTDA [38]	2-stage	Lidar + img	0.08	76.65	72.86	64.51
MV3D [25]	2-stage	Lidar + img	0.36	86.02	76.90	68.49
PIXOR [32]	1-stage	Lidar	0.09	86.79	80.75	70.60
Proposed	1-stage	Lidar + img	0.09	89.01	84.69	78.71
